# Comparative stigmatic transcriptomics reveals self and cross pollination responses to heteromorphic incompatibility in *Plumbago auriculata* Lam.

**DOI:** 10.3389/fgene.2024.1372644

**Published:** 2024-03-06

**Authors:** Di Hu, Di Lin, Shouli Yi, Suping Gao, Ting Lei, Wenji Li, Tingdan Xu

**Affiliations:** ^1^ College of Fine Art and Calligraphy, Sichuan Normal University, Chengdu, China; ^2^ Sichuan Certification and Accreditation Association, Chengdu, China; ^3^ College of Landscape Architecture, Sichuan Agricultural University, Chengdu, China; ^4^ School of Design, Chongqing Industry Polytechnic College, Chongqing, China

**Keywords:** heteromorphic self-incompatibility, pollination, pollen-stigma interaction, transcriptome, *Plumbago auriculata* Lam

## Abstract

“Heteromorphic self-incompatibility” (HetSI) in plants is a mechanism of defense to avoid self-pollination and promote outcrossing. However, the molecular mechanism underlying HetSI remains largely unknown. In this study, RNA-seq was conducted to explore the molecular mechanisms underlying self-compatible (SC, “T × P” and “P × T”) and self-incompatible (SI, “T × T” and “P × P”) pollination in the two types of flowers of *Plumbago auriculata* Lam. which is a representative HetSI plant. By comparing “T × P” vs. “T × T”, 3773 (1407 upregulated and 2366 downregulated) differentially expressed genes (DEGs) were identified, 1261 DEGs between “P × T” and “P × P” (502 upregulated and 759 downregulated). The processes in which these DEGs were significantly enriched were “MAPK (Mitogen-Activated Protein Kinases-plant) signaling pathway”, “plant-pathogen interaction”,“plant hormone signal transduction”, and “pentose and glucuronate interconversion” pathways. Surprisingly, we discovered that under various pollination conditions, multiple notable genes that may be involved in HetSI exhibited distinct regulation. We can infer that the HetSI strategy might be unique in *P. auriculata*. It was similar to “sporophytic self-incompatibility” (SSI) but the HetSI mechanisms in pin and thrum flowers are diverse. In this study, new hypotheses and inferences were proposed, which can provide a reference for crop production and breeding.

## 1 Introduction

Mate selection is a critical factor for ensuring successful reproduction, with self-incompatibility (SI) being one of the predominant mate selection strategies in plants. SI plays a crucial role in promoting out-crossing to achieve genetic diversity by rejecting self-pollen (Lian et al., 2021). SI can be classified into two categories: homomorphic and heteromorphic. Homomorphic SI (HomSI) plants have only one type of flower, while heteromorphic SI (HetSI) plants have distyly or tristyly flowers (Jain et al., 2023). SI plants have developed two systems to prevent incorrect pollen deposition: sporophytic self-incompatibility (SSI) and gametophytic self-incompatibility (GSI) (Wang et al., 2023). Based on the S genotype of the sporophyte, the SSI is determined, pollen germination failure occurs on the stigma’s surface, preventing the pollen from penetrating the style tissue (Pauline et al., 2023). In GSI, haploid pollen itself determines the phenotype, and pollen tube growth can be arrested before entering the ovary (Sassa, 2016; Pauline et al., 2023). Notably, GSI systems and homomorphic systems have been studied more extensively than heteromorphic systems (McCubbin, 2008). Studies have reported that in Solanaceae, Rosaceae and Plantaginaceae, GSI is based on the *SLF* (*SFB*)/*SRNase* system, and in Brassicaceae SSI is based on the *SP11* (*SCR*)/*SRK* system (Lv et al., 2022; Goring et al., 2023). Significant advancements have been made in comprehending the molecular foundation of HetSI, particularly in *Primula* and *Fagopyrum* (Banović et al., 2010; Li et al., 2023). Studies have demonstrated that HetSI in both types of plants shares the same HetSI controller-S locus and exhibits similar pollen‒stigma interactions (Matsui and Yasui, 2020).

**FIGURE 1:**
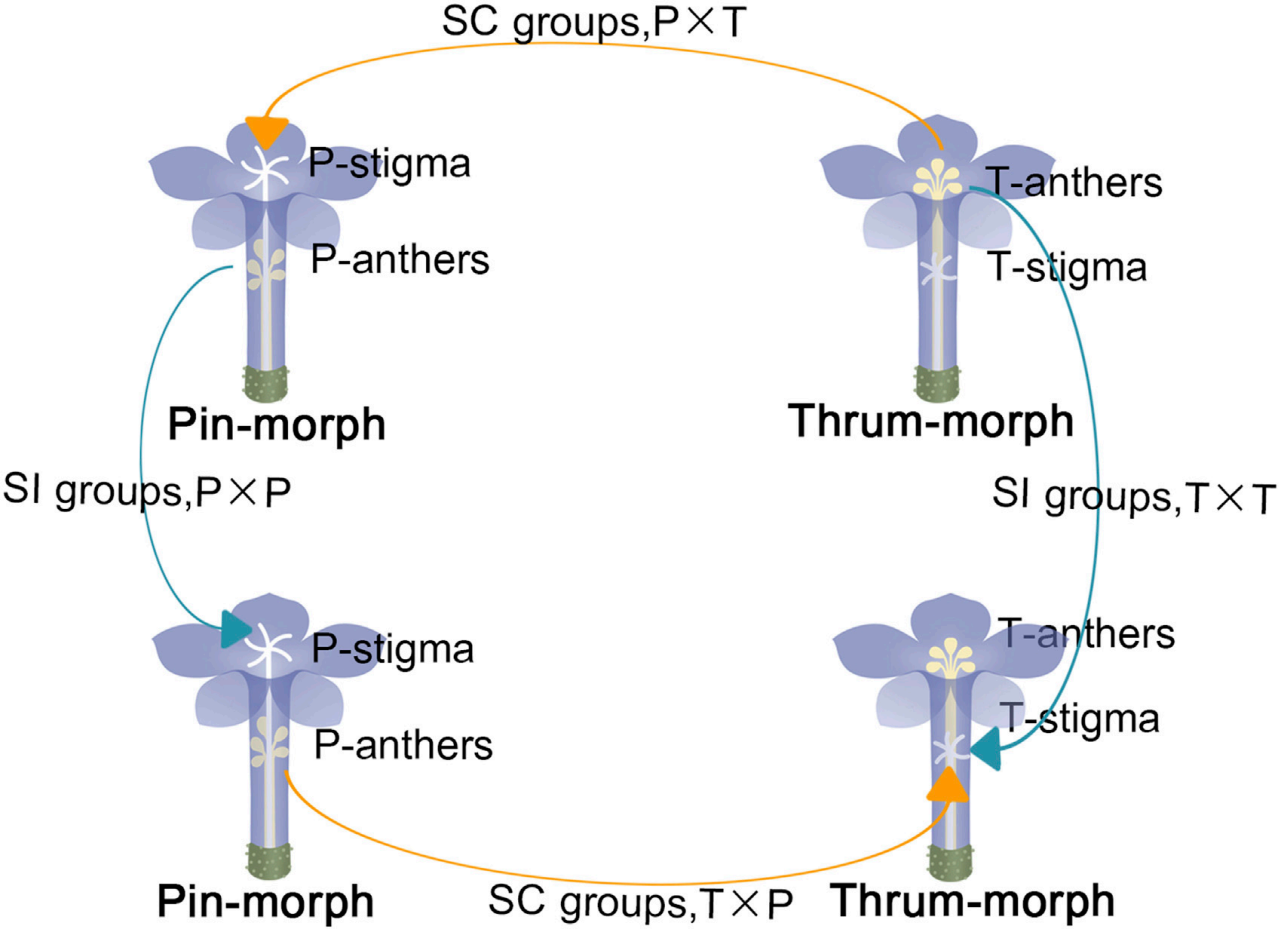
“Thrum-morph flower” (T) and “pin-morph flower” (P). the connotation of “P × T”: pin-morph stigmas that are pollinated with thrum pollens; the connotation of “P × P”: pin-morph stigmas that are pollinated with pin pollens; the connotation of “T × P”: thrum-morph stigmas pollinated with pin pollens; the connotation of “T × T”: thrum-morph stigmas pollinated with thrum pollens; “Self-incompatible (SI) pollinations” are shown by the arrows of blue color, and “self-compatible (SC) pollinations” are indicated by orange arrows.

A frequently encountered HetSI plant with dry stigmas is *Plumbago auriculata* Lam. (Plumbaginaceae) (Hu et al., 2019). The Plumbaginaceae family comprises approximately 650–1000 species, with a significant number of them being HetSI plants (Mucina and Hammer, 2019). These plants serve as excellent research systems for HetSI studies. Through artificial pollination, we found that once pollination occurs with compatible pollen, a series of signaling events are triggered, including pollen adhesion, germination, pollen tube elongation, and fertilization, all of which occur within 4 h. However, no pollen tubes were discovered in SI pollinations (Hu et al., 2019). The metabolomic study revealed that mature styles stored abundant nutrients required for the energy-consuming process in the pollen tube growth. Despite the failure of fertilization in SI pollinations, the styles still contained sufficient energy reserves to support the growth of other SC (self-compatible) pollen grains (Figure 1). Moreover, the morphology and metabolome of SI in pin and thrum flower styles were found to be distinct (Hu et al., 2019; Hu et al., 2020). This led us to hypothesize that different flower types may have varying mechanisms for SI. However, data concerning the SI reaction at the molecular level in *P. auriculata* are still very scarce. To gain a deeper understanding of the *P. auriculata* HetSI mechanism at the genomic level, RNA-seq analysis was conducted on stigmas after different pollination events. This study’s transcriptome analysis offers valuable insights into the potential molecular mechanisms influencing *P. auriculata* HetSI and aims to provide new evidance that support the understanding of the interaction between the pollen and stigma in HetSI.

## 2 Materials and approaches

### 2.1 Plant material and the environment of growth

Pin and thrum flowers of *P. auriculata* were cultivated separately in the greenhouse at Sichuan Normal University, located in Sichuan, China. The day before dehiscence, floral buds of pollen receptor flowers were chosen at random, emasculated, and bagged.

At 8:00 a.m., pistils were manually pollinated with SC pollination: “P × T” (pin pistils inter - pollinated with thrum pollen), “T × P” (thrum pistils inter - pollinated with pin pollen) and SI pollination: “T × T” (thrum pistils intra - pollinated with thrum pollen), “P × P” (pin pistils intra - pollinated with pin pollen). They were collected 2 h later, at 10:00 a.m. (Hu et al., 2019; Hu et al., 2020). Fifty milligrams of each sample was processed for analysis (Figure 1). For every sample, three biological replicates were employed. Upon instant freezing in liquid nitrogen, samples were kept at the temperature of −80 °C in order to extract RNA.

### 2.2 RNA-seq and data analyses

With a “RNAprep Pure Plant Kit” (Tiangen, China) and “TRlzol Reagent” (Life Technologies, United States), from every sample, entire RNA was extracted. Using an “Agilent 2100 Bioanalyzer” (Agilent Technologies, United States) and “Nanodrop 2000” (Thermo Fisher Scientific, United States), RNA’s quality was checked. The “Hieff NGS Ultima Dual-mode mRNA Library Prep Kit for Illumina” (Yeasen Biotechnology, China) was used to construct the cDNA library following the manufacturer’s proposals. Utilizing a “NovaSeq6000” (Illumina, United States), the cDNA libraries were sequenced. Finally, clean data were obtained through “in-house Perl scripts”. Sequence duplication, GC content, Q20, and Q30 values were calculated.

For the sake of determining the pertinent expression standards of SI and SC pollinations, the “DESeq R package” (1.10.1) was utilized. “The false discovery rate” (FDR) was controlled for the resultant “*p* values” by applying Benjamini and Hochberg (1995). Genes that DESeq identified as differentially expressed (DEGs) have an adjusted *p*-value <0.05.

Functional enrichment for the DEGs was accessed through the following databases: KOG/COG/eggNOG (Clusters of Orthologous Groups of proteins), NR (NCBI nonredundant protein sequences), Pfam (Protein family), KEGG (Kyoto Encyclopedia of Genes and Genomes), Swiss-Prot (Swiss-Prot protein sequence database), and GO (Gene Ontology) enrichment analysis.

### 2.3 RT-qPCR analysis

A total of 15 DEGs were analyzed by RT‒qPCR to verify the dependability of RNA-seq. The “TUREscript 1^st^ Stand cDNA SYNTHESIS Kit” (Aidlab, China) was used for first-stand cDNA synthesis. By utilizing a “qTOWER 2.2 Real-Time Thermocyclers” (Analytik Jena, GER.) with three biological replicates, RT‒qPCR was performed. With the *GAPDH* gene serving as an internal control, the 2^−ΔΔCT^ method was used to calculate the relative quantification of gene expression (Livak and Schmittgen, 2001).

## 3 Results

### 3.1 Stigmatic comparative transcriptomics of HetSI

For the purpose of exploring the molecular mechanisms underlying interactions in pollen-stigma of *P. auriculata*, SC group (“T × P” and “P × T”) and SI group (“P × P” and “T × T”) pollinated styles were collected, and “Illumina NovaSeq technology” was employed to investigate the stigma transcriptome. Per sample from transcriptome sequencing generated 6.00 Gb of clean data, with a “Q-score” larger than the Q30 value in more than 93.85% of cases. Furthermore, every sample’s mapping ratio against the reference genome with the scope from 78.89% to 82.48%, as the Table 1 revealed. The data demonstrated robust sequencing and mapping results in our study. After assembly, we extracted 85249 unigenes, 44407 of which were annotated in the reference genome. In addition, we used the “*R* ^2^ value” to evaluate the repeat correlation, which showed a high intragroup similarity.

**TABLE 1:** The sequencing data from RNA-seq analysis of different (“P × P”, “P × T”, “T × T” & “T × P”) styles of pollination.

Sample-ID	Read number	Base number	GC content (%)	%≥Q30	Mapped reads	Mapped ratio (%)
P × P1	20,241,269	6,051,617,888	47.15	94.33	16,272,607	80.39
P × P2	21,371,047	6,383,933,324	46.82	94.48	17,118,064	80.10
P × P3	20,903,182	6,249,338,828	46.85	94.34	16,539,064	79.12
P × T1	27,219,609	8,135,444,050	46.91	94.95	21,671,239	79.62
P × T2	21,343,126	6,378,384,660	46.99	94.08	17,043,586	79.86
P × T3	20,561,578	6,145,049,000	46.84	93.93	16,220,265	78.89
T × P1	23,853,189	7,129,987,150	47.18	94.30	19,390,489	81.29
T × P2	21,318,941	6,372,725,284	47.68	94.84	17,583,650	82.48
T × P3	20,110,248	6,015,229,234	47.21	94.10	16,518,736	82.14
T × T1	20,069,489	5,999,413,952	47.18	93.85	16,111,513	80.28
T × T2	23,303,767	6,964,945,966	47.28	94.26	18,705,649	80.27
T × T3	23,175,587	6,928,609,824	47.35	93.95	18,502,888	79.84

### 3.2 DEGs in stigmas of different pollination

**FIGURE 2:**
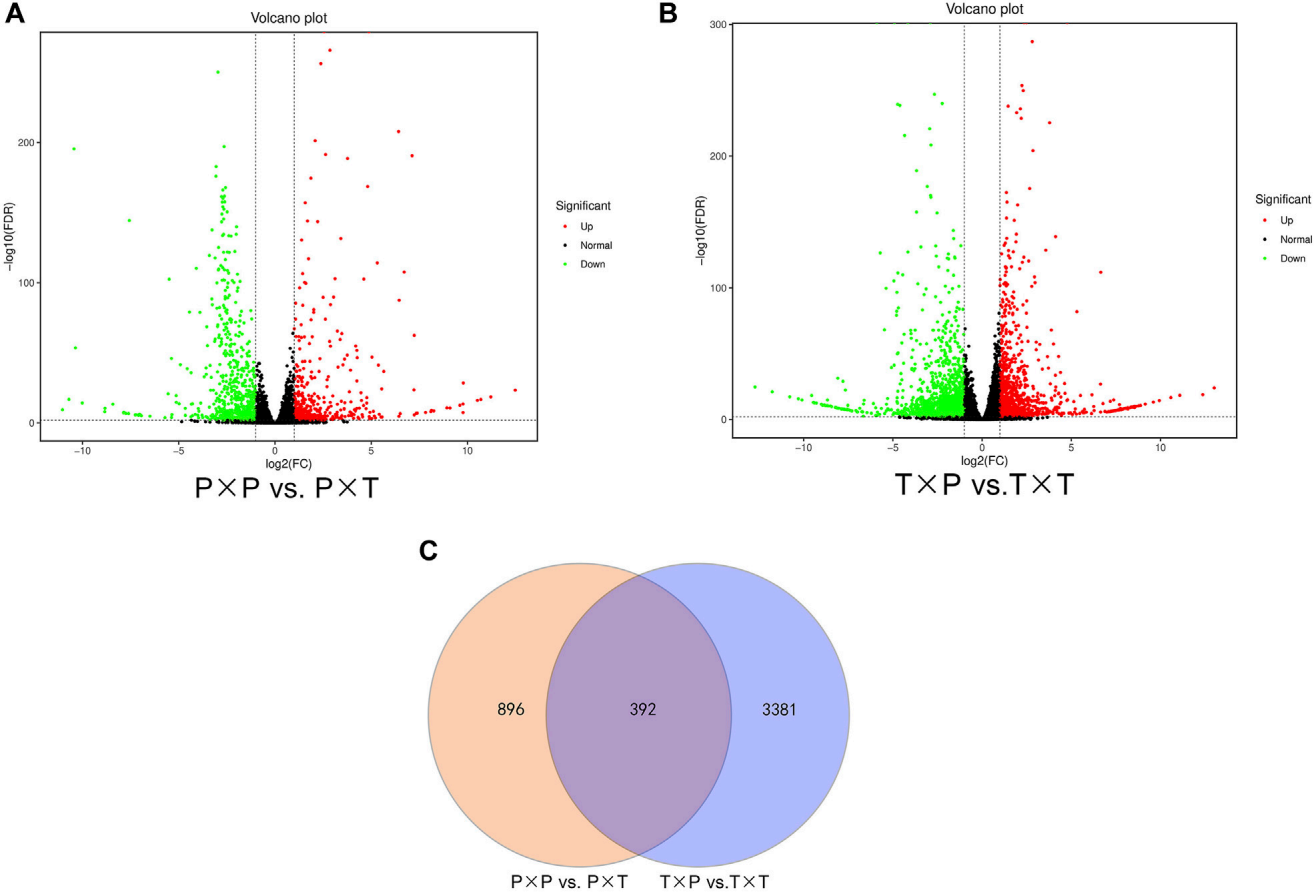
Statistics of DEGs in different pollination **(A,B)** Volcano plot of the identified DEGs in comparing “T × P” vs. “T × T” as well as “P × T” vs. “P × P”; **(C)** Venn diagrams of common and specific DEGs between “T × P” vs. “T × T” and “P × T” vs. “P × P”.

Fold change (FC) > 2 as well as FDR <0.01 were employed to quantify the transcript expression and identify DEGs. The comparison of “T × P” vs. “T × T” generated 3773 (1407 upregulated and 2366 downregulated) DEGs. There were 1261 DEGs between “P × T” and “P × P” (502 upregulated and 759 downregulated) DEGs (Figures 2A, B). Among them, 392 DEGs were shared by both comparison groups (Figure 2C).

### 3.3 Gene enrichment analysis for pollination of different flower types

Within the COG, KOG, Nr, Swiss-Prot, Pfam and eggNOG databases, 1173, 1882, 3303, 2723, 2791, and 2924 DEGs were annotated successfully in the context of a comparison between “T × P” vs. “T × T”, and the results showed many DEGs classified in the transporting process of the carbohydrate and metabolism and signal transduction mechanisms. Additionally, protein turnover, posttranslational modification, chaperons and lipid transport revealed a great number of genes in the KOG and COG databases. For “P × T” and “P × P” (COG: 418; KOG: 618; Nr: 1121; Swiss-Prot: 916; Pfam: 983; eggNOG: 996), DEGs were enriched in the classifications above as well (Table 2; Supplementary Figure S1).

**TABLE 2:** Functional enrichment analysis of all DEGs by GO, COG, KOG, KEGG, NR, Pfam, eggnog, and Swiss-Prot.

Compare	Annotated	GO	KEGG	COG	KOG	Swiss-Prot	NR	Pfam	eggnog
P × T vs. P × P	1,133	801	855	418	618	916	1,121	983	996
T × P vs. T × T	3,325	2,377	2,508	1,173	1,882	2,723	3,303	2,791	2,924

**FIGURE 3:**
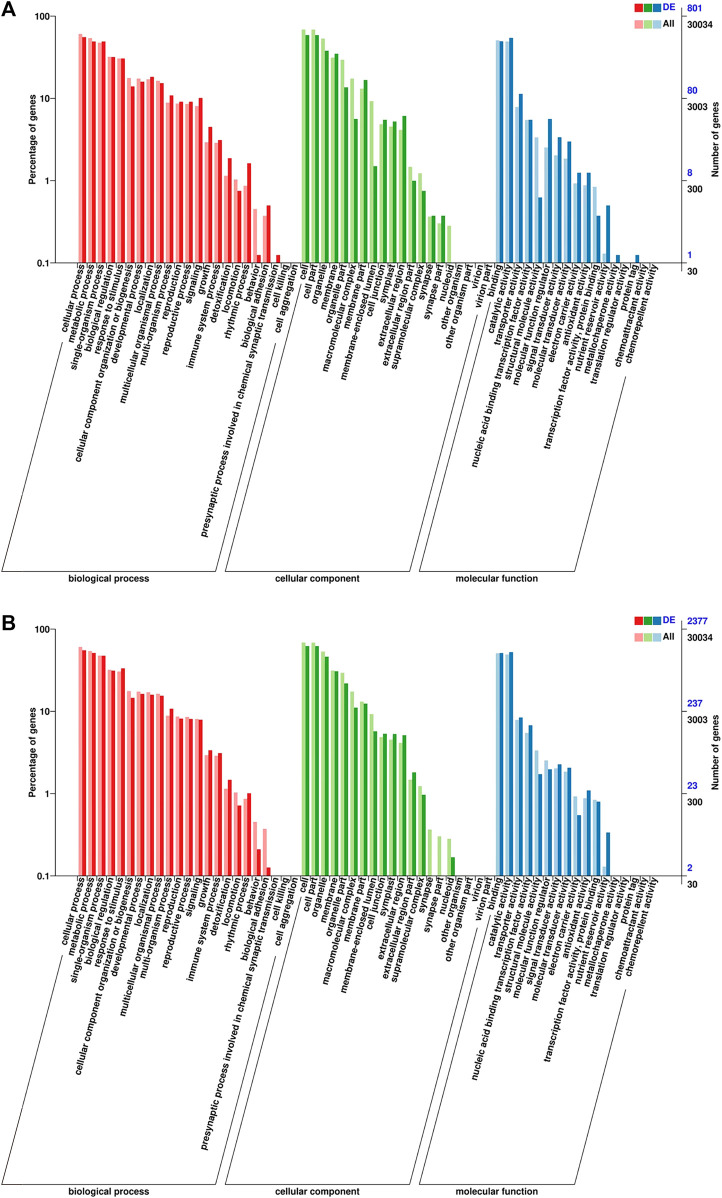
The GO terms classification of DEGs in **(A)** “P × T” vs. “P × P” and **(B)** “T × P” vs. “T × T”.

To further analyze the function of the DEGs, we annotated the DEGs using “Gene Ontology” (GO) for analysis. The DEGs were categorized into the following GO primary functions, namely, “molecular function” (MF), “biological process” (BP), and “cellular component” (CC). In total, 2377 and 801 DEGs were assigned GO annotations in the “T × P” vs. “T × T” and “P × T” vs. “P × P” comparison groups, respectively (Figure 3; Supplementary Table S1). For BP, the three most abundant terms in all groups were cellular process (1312 and 445) (numbers in parentheses represent the number of unigenes in “T × P” vs. “T × T” and “P × T” vs. “P × P”, respectively), metabolic process (1218 and 393) and single-organism process (1127 and 393). For CC, these terms were cell (1477 and 471), cell part (1477 and 471) and organelle (1096 and 304). For MF, the terms were binding (1217 and 396), catalytic activity (1247 and 436), and transporter activity (200 and 91) (Table 3). In the “T × P” vs. “T × T” comparison group, the two highest secondary functions of BP were the multiorganism process and response to external stimulus. Cell periphery and protein kinase activity were abundant in CC and MF, respectively. In “P × T” vs. “P × P”, the most overrepresented GO terms in BP were protein phosphorylation and transmembrane transport, and the terms were membrane in CC and protein kinase activity in MF (Supplementary Figure S2).

**TABLE 3:** The number of total genes and DEGs of GO terms in “P × T” vs. “P × P” and “T × P” vs. “T × T”.

Comparison	GO annotations	GO classification	All genes	DEGs
P × T vs. P × P	cellular component	organelle	16056	304
		cell part	20603	471
		cell	20606	471
	molecular function	transporter activity	2366	91
		catalytic activity	14772	436
		binding	15287	396
	biological process	single-organism process	14317	393
		metabolic process	16257	393
		cellular process	18196	445
T × P vs. T × T	cellular component	organelle	16056	1096
		cell part	20603	1477
		cell	20606	1477
	molecular function	transporter activity	2366	200
		catalytic activity	14772	1247
		binding	15287	1217
	biological process	single-organism process	14317	1127
		metabolic process	16257	1218
		cellular process	18196	1312

To investigate the effect of DEGs on pathways in more detail, based on the KEGG database, we also carried out pathway enrichment analysis of DEGs. According to the results for both upregulated genes of the “T × P” vs. “T × T” comparison group, DEGs were gathered in the “transduction of plant hormone signal”, “plant-pathogen interaction”, “protein processing in endoplasmic reticulum”, “endocytosis” and “starch and sucrose metabolism”. For downregulated genes, those containing the greatest number were “plant‒pathogen interaction”, “MAPK (plant) signaling pathway”, “pentose and glucuronate interconversions”, “plant hormone signal transduction” and “starch and sucrose metabolism”. For “P × T” vs. “P × P”, among the upregulated genes were “MAPK (plant) signaling pathway”, “plant-pathogen interaction” and “plant hormone signal transduction”. The processes in which downregulated genes were enriched were “pentose and glucuronate interconversions”, “plant-pathogen interaction” and “metabolism of starch and sucrose” as showcased in the Table 4.

**TABLE 4:** Enrichment of up- and downregulated DEGs in specific KEGG pathways among stigmas of different *P. auriculata* pollination.

Group	Regulated	KEGG pathway	Ko ID	DEGs in pathway	All genes in pathway
P × T vs. P × P	Up	Plant hormone signal transduction	ko04075	18	779
		MAPK (plant) signalling pathway	ko04016	19	540
		Plant‒pathogen interaction	ko04626	26	950
	Down	Plant hormone signal transduction	ko04075	11	779
		Phenylpropanoid biosynthesis	ko00940	11	352
		Starch and sucrose metabolism	ko00500	16	586
		Plant‒pathogen interaction	ko04626	26	950
		Pentose and glucuronate interconversions	ko00040	31	349
T × P vs. T × T	Up	Endocytosis	ko04144	20	617
		Starch and sucrose metabolism	ko00500	21	586
		Protein processing in endoplasmic reticulum	ko04141	25	733
		Plant hormone signal transduction	ko04075	28	779
		Plant‒pathogen interaction	ko04626	35	950
	Down	MAPK (plant) signalling pathway	ko04016	42	540
		Starch and sucrose metabolism	ko00500	42	586
		Plant hormone signal transduction	ko04075	43	779
		Pentose and glucuronate interconversions	ko00040	44	349
		Plant‒pathogen interaction	ko04626	82	950

### 3.4 Identification of genes involved in self-incompatibility

To obtain further insights, all genes were categorized based on the GO or KEGG databases. Notably, 10 genes were annotated to the plant *SI S1* (self-incompatibility protein S1), while 3 genes were annotated to *SRK* with the full name of “S-locus receptor kinase”, *PSEUDOSRKA* with the full name of “putative inactive G-type lectin S-receptor-like serine/threonine-protein kinase SRK” as the major determinants of SI. *ARC1* (Arm repeat containing 1), *THL1/2* (thioredoxin H-like), *Exo70A1* (Exocyst compounds), *MLPK* (M-locus protein kinase), and *MOD* (Aquaporin) were also found as the downstream regulators of SI. *MPK3* (Mitogen-activated protein kinase 3) and *MKK2/3* (Mitogen-activated protein kinase kinase 2/3) which belonged to MAPK family, were interacting with *FERON* (Receptor-like protein kinase FERONIA) to regulate pollen receptivity. *OFT23* (O-fucosyltransferase 23) and *KCS6* (3-ketoacyl-CoA synthase 6) involved in pollen-stigma interaction; *BABL* (Basic blue protein), *FIMB5* (Fimbrin-5), *PLRX4*, namely “Pollen-specific leucine-rich repeat extensin-like protein 4”, *TFIIB1* (Transcription initiation factor IIB) and *AL11A* (Pectin Methylesterases) were regulators of pollen tube growth (Table 5).

**TABLE 5:** Self-incompability related candidates, function, and localization.

Gene name	Gene description	Subcellular localization
*Self-incomp-S1*	Exhibits specific pollen self-inhibitory activity thus preventing self-fertilization (Miao et al., 2015)	Accumulates in the stigma
*SRK*	Female specificity determinant of self-incompatibility (Goring et al., 2023)	Accumulates in the stigma
*SRK6*	Involved in sporophytic self-incompatibility system, acting in combination with S-locus-specific glycoproteins (Goring et al., 2023)	Accumulates in the stigma
*PSEUDOSRKA*	Truncated and inactivated form of SRK (Fujii et al., 2020)	Accumulates in the stigma
*ARC1(SD17)*	A novel U-box protein involved in the rejection of self-incompatible pollen (Abhinandan et al., 2023)	Accumulates in the stigma
*MLPK (RLCK VII)*	A positive effector of the SI and directly interacts with SRK (Kakita et al., 2007a)	Accumulates in the stigma
*THL1/2(TRX1/2)*	Interact with the SRK kinase domain and inhibit basal SRK activity prior to the arrival of SI pollen (Cabrillac et al., 2001)	Accumulates in the stigma
*MPK3*	MAPK family which is required for maintaining stigma receptivity to accept compatible pollen (Jamshed et al., 2020)	Accumulates in the stigma
*MKK2/3*		
*FERON (FER)*	A stigmatic ROS regulator, involved in pollen-pistil interaction (Huang et al., 2023)	Accumulates in the stigma
*Exo70A1*	A factor regulates pollen hydration and germination (Jamshed et al., 2020)	Accumulates in the stigma
*MOD*	Regulator of water transportation between pollen and stigma in SI (Maurel et al., 2008)	Accumulates in the stigma
*BABL (ARPN)*	A factor regulates pollen tube growth (Zhang et al., 2021a)	Accumulates in the stigma
*KCS6 (CUT1)*	Involved in accumulation of pollen coat lipid and pollen hydration (Zhang et al., 2021b)	Accumulates in pollen grains
*OFT23 (OFUT23)*	Facilitates pollen tube penetration through the stigma-pistil interface (Smith et al., 2018)	Accumulates in pollen grains
*FIMB5 (FIM5)*	Involved in the regulation of pollen germination and tube growth (Baumberger et al., 2003; Wu et al., 2010; Zhou et al., 2013; Tang et al., 2020)	Accumulates in pollen grains
*PLRX4 (PEX4)*		
*TFIIB1*		
*AL11A*		

### 3.5 RNA-seq data by RT‒qPCR Confirmation

**FIGURE 4:**
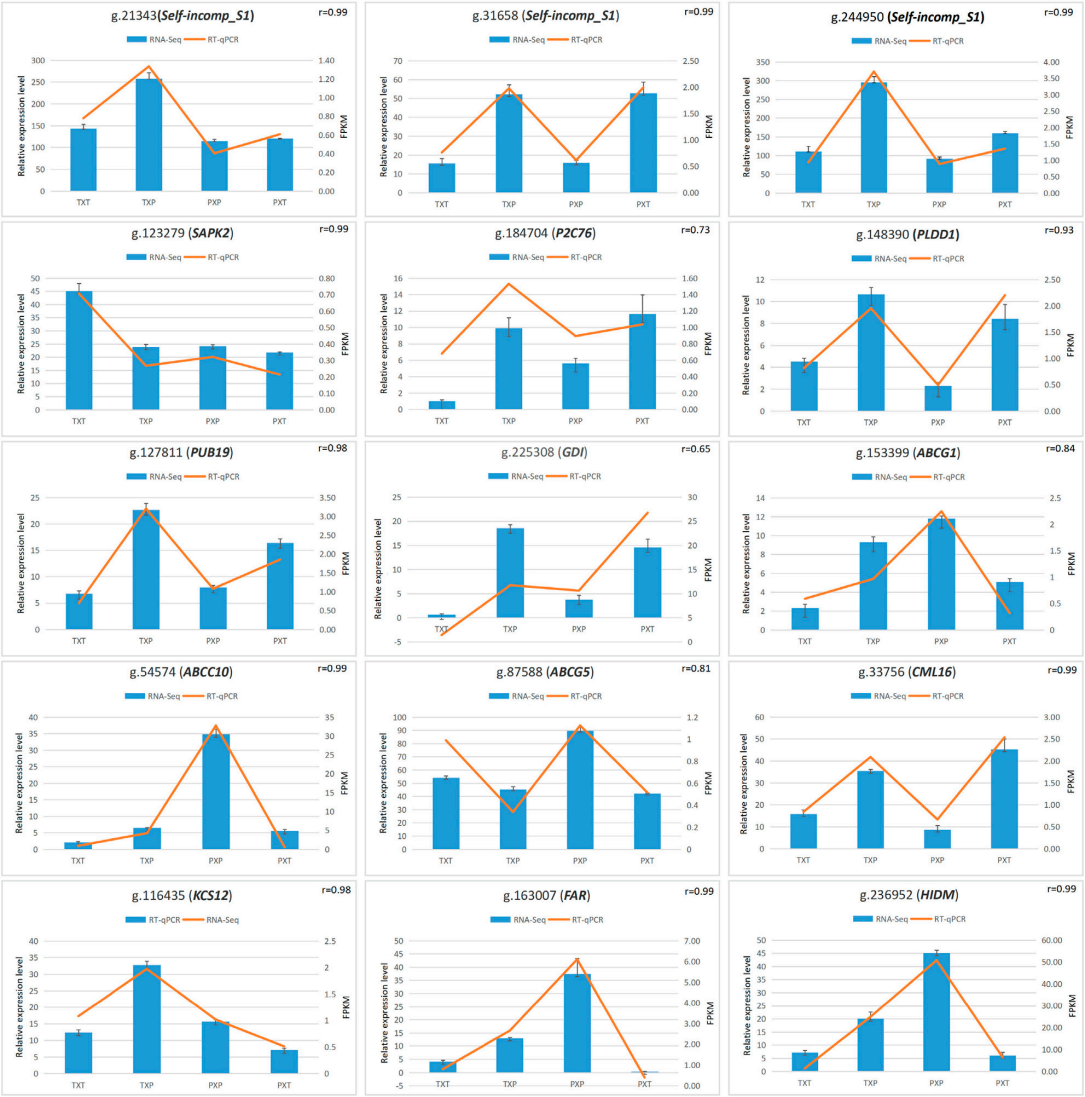
The correlation analysis of RT‒qPCR values and FPKM values to verify the RNA-seq data.

It was RT‒qPCR that was conducted to verify DEGs’ expression. These selected DEGs were pertinent to self-incompatibility (g.21343, *Self-incomp-S1*; g.244950, *Self-incomp-S1*; g.31658, *Self-incomp-S1*), signal transduction (g.184704, *P2C76*; g.127811, *PUB19*; g.148390, *PLDD1*; g.225308, *GDI*; g.123279, *SAPK2*), ABC transporters (g.153399, *ABCG1*; g.54574, *ABCC10*; g.87588, *ABCG5*), plant‒pathogen interaction (g.33756, *CML16*; g.116435, *KCS12*) and lipid transport and metabolism (g.163007, *FAR*; g.236952, *HIDM*) (Supplementary Table S2). The expression level and correlation analysis showed a consistent trend between RNA-seq and RT‒qPCR analysis (Figure 4).

## 4 Discussion

### 4.1 S-locus-related genes in HetSI

Studies of crucifers (SSI) have revealed that the S-locus regulates SSI identification, and over a hundred S-haplotypes were present in *Brassica napus.* It is believed that the SRK-SCR recognition via phosphorylation and degradation facilitates the pollen‒pistil interaction (Gu et al., 1998; Goring et al., 2014) and the SI signal transducers include SRK, SLG, SCR/SP11, and MLPK (Takasaki et al., 2000; Takayama et al., 2000; Murase et al., 2004; Kakita et al., 2007b; Kitashiba and Nasrallah, 2014). Nevertheless, SRK-ARC1 interaction was not functioning in *Corylus*, indicating that the SSI system was distinct between *Corylus* and *Brassica* (Hou et al., 2022). Additionally, the *Fagopyrum* genome did not contain *SRK* or *SLG* (Banović et al., 2010). The following SSI component genes were identified in this HetSI study: *SRK*, *SRK6*, *PSEUDOSRKA*, *ARC1*, *MLPK*, *THL1/2*, and *Exo70A1*. Furthermore, *ARC1*, *MLPK*, *THL1/2*, *Exo70A1*, and *MOD* were also intimately associated with the S-locus. It was speculated that the SI signal in *Brassica* was transmitted via the “SRK-ARC1-Exo70A1” pathway (Wang et al., 2023). With the fall of self-pollen, SRK and SP11/SCR engaged to generate a “heterotetrameric assembly” (2:2 eSRK: SP11/SCR), and ARC1 interacted with the SRK and the domains of MLPK kinase to regulate SI through phosphorylation (Abhinandan et al., 2023). Subsequently, phosphorylated ARC1 is linked to Exo70A1 (Goring et al., 2014; Doucet et al., 2016). To prevent pollen hydration, the “ACR1-Exo70A1 complex” undergoes translocation and degradation at the “26S proteasome”, which causes the channels of MOD to open. (Maurel et al., 2008; Wang et al., 2023). Serving as a potential inhibitor to prevent spontaneous activation, THL1/2 interacted with SRK (Cabrillac et al., 2001). It is conceivable that the mechanism in HetSI might be similar to SSI. Remarkably, SI S1 was exclusively detected in GSI plants such as *Citrus* and *Papaver* (Miao et al., 2015; Goring et al., 2023). This is the first HetSI research of its kind. In the GSI study, the SI S1 protein could significantly inhibit the pollen germination frequency (Miao et al., 2015). Besides, the SI S1 protein of *Citrus* was upregulated after self-pollination, whereas its expression remained relatively low following cross-pollination. According to the findings, SI S1 was downregulated after SI pollination in both thrum and pin types, which was an opposite trend to that of *Citrus* (Miao et al., 2015). The results suggest that SI S1 may exhibit diverse role in the HetSI mechanism of *P. auriculata*. After recognition by the “SRK-ARC1-Exo70A1” pathway, MAPKs, in conjunction with *FERON*, determined pollen receptivity by regulating the ROS level on the stigma surface (Huang et al., 2023). *KCS6* was involved in the accumulation of pollen coat and affected water transfer between pollen and stigma, which afterward regulated pollen hydration (Zhang et al., 2021b). In *Arabidopsis*, *OFT23* may promote pollen tube penetration (Smith et al., 2018). *BABL*, *FIMB5*, *PLRX4*, *TFIIB1*, and *AL11A* were also identified as candidates for HetSI (Table 5) and previously reported as regulators of pollen tube formation (Baumberger et al., 2003; Wu et al., 2010; Zhou et al., 2013; Tang et al., 2020; Zhang et al., 2021b).

Furthermore, in addition to coding RNAs (mRNAs), non-coding RNAs (ncRNAs) have been identified as playing multifaceted roles in pollen-stigma recognition. In *Brassica*, a trans-acting small non-coding RNA (sRNA) *Smi* (SP11 methylation inducer), has been implicated in facilitating the SRK-SP11 interaction through specific promoter methylation (Finnegan et al., 2011). MicroRNAs (miRNAs), specifically 18-nt miRNAs (novel_miR_49), have been recognized as potential candidates influencing SI in blueberry (*Vaccinium* sp.). Additionally, miRNAs associated with phytohormone signal transduction have been proposed to play a role in pollen-stigma interactions in SI (Yang et al., 2022). Furthermore, overexpression of the long non-coding RNA (lncRNA) bra-miR5718HG has been shown to inhibit pollen tube growth in *Brassica campestris* (Shi et al., 2024). However, researches in this field remains largely in its infancy and requires further studies to achieve a more comprehensive understanding of SI mechanisms.

### 4.2 Different flower types might have varying SI mechanisms

Our previous research demonstrated that HetSI in *P. auriculata* is characterized by a strict rejection of SI pollen by the dry-type stigma, where no pollen tubes penetrate the papillae. Notably, 2 hours post SC pollination, pollen-stigma cross-linking was evident, with distinct morphological differences observed between the crosses “P × T” and “T × P”. In “T × P”, a “foot” structure penetrated the stigma, unlike in “P × T”, where germinated pollen adhered to the stigma without forming a “foot” structure, and the surrounding papillae became less globular (Hu et al., 2019). Additionally, SC pollen exhibited a faster growth rate on the thrum morphotype compared to the pin morphotype. Metabolomic analyses revealed metabolic variations in the SI responses of pin and thrum flowers (Hu et al., 2019; 2020).

Through RNA-seq and RT‒qPCR, we explored HetSI mechanisms, finding a higher quantity of DEGs in “T × P” vs. “T × T” than in “P × T” vs. “P × P”, suggesting a more complex HetSI mechanism in the former. This indicates that the cross-direction between pin and thrum flowers may influence pollen recognition and germination, with thrum flowers potentially exhibiting a more complex and rapid germination process to offset the pollination disadvantages posed by their lower style position. Furthermore, in GSI studies, MAPK signaling was shown to induce programmed cell death (PCD) to irreversibly inhibit SI pollen (Zhou et al., 2020), whereas in sporophytic self-incompatibility (SSI) research, MAPKs were essential for maintaining stigma receptivity to compatible pollen. Specifically, MPK3/4 and MKK2/3 phosphorylate Exo70A1, localizing it at plasma membranes to regulate exocytosis and facilitate SC pollination in *Arabidopsis* (Jamshed et al., 2020). In our study, DEGs in the MAPK (plant) signalling pathway were downregulated in “T × P” vs. “T × T” but exhibited the opposite trend in“P × T” vs. “P × P” (Table 4). The identification of female S determinants, such as SRK in *Brassica* and *Arabidopsis* for SSI, and SI S1 in *Papaver* for GSI, along with critical components like *PGAP1,* in our results (Goring et al., 2023), suggests that *P. auriculata* combines both SSI and GSI characteristics, similar to another HetSI species, *Primula maximowiczii* (Lu et al., 2018). HetSI in pin flowers may share pathways with *Arabidopsis* SSI, where decreased MAPKs in “T × P” vs. “T × T” could inhibit MAPKs - Exo70A1 interaction and reduce pistil receptivity. Conversely, in thrum flowers, resembling GSI in *Papaver* (Goring et al., 2023)*,* the opposite trend of MAPKs in “P × T” vs. “P × P” could trigger PCD in the stigma and facilitate HetSI.

### 4.3 Pathogen–plant interaction related to pollen‒stigma recognition

Comparing SC with SI, most of the DEGs were detected in the “plant‒pathogen interaction”, “plant hormone signal transduction”, “MAPK (plant) signaling”, and “pentose and glucuronate interconversion” pathways. Pathogen–plant interactions are involved in the plant immune system, and entailed specific cell-cell recognition processes that selectively inhibit certain pathogens while facilitating others (Muñoz-Sanz et al., 2020). Two contrasting interaction patterns were included in pathogen-plant interactions that share a similar mechanism with pollen-pistil interactions in SI, and defence-related genes function in both pathogen and SI pollen. SRK-like S-domain RLKs have been implicated in pathogen resistance, recognizing extrinsic ligands (Sanabria et al., 2008; Catanzariti et al., 2015). Numerous stigma-specific genes encode proteins for stress and defence, according to studies in *Brassica* and *Oryza sativa*. Additionally, some common cis-regulatory elements of stigma-specific genes are shared with stress-responsive genes (Li et al., 2007). In our comparative group, 950 genes were enriched in the “pathogen–plant pathway”, second only to “carbon metabolism”. A total of 866 genes were in the “defence response (immune system process) pathway”, which was related to GO terms (Table 4; Supplementary Table S3). The results support the hypothesis that pathogen-plant and pollen-stigma interactions might involve some common signalling pathways.

### 4.4 Plant hormone signal transduction in HetSI

A vital component of *P*. *auriculata* pollination is plant hormone signal transduction. Brassinosteroids (BRs) were previously demonstrated to be important phytohormones for reproductive processes in plants (Li and He, 2020). Research on *Primula* has revealed that female self-incompatibility is influenced by the brassinosteroid-inactivating enzyme, cytochrome P450 *CYP734A50* (Huu et al., 2022). To enhance self-fertilization, loss of independent function mutations in *CYP734A50* and exogenous BR treatment altered both female compatibility and style morphology (Huu et al., 2022). Consequently, it has been concluded that female self-incompatibility in *Primula* is mediated by the maintenance of low brassinosteroid (BR) levels within the style (Huu et al., 2022). In our study, *CYP92A6* (Typhasterol) in the brassinosteroid biosynthesis pathway decreased sharply in both the “T × P” vs. “T × T” and “P × T” vs. “P × P” comparisons, and *CYP85A1* showed the same trend in “T × P” vs. “T × T”, indicating that BR decreased in styles to modify the mate recognition of SI (Supplementary Figure S3). *ARF* (Auxin response factor), *EIN* (Ethylene-insensitive protein) and *ABF 2* (Abscisic acid-insensitive 5-like protein 5) overexpression indicated that auxin, ethylene and abscisic acid signalling may participate in HetSI. Auxin is likely to participate in pollen-pistile recognition and pollen tube growth (Matsuda et al., 2015; Zhao et al., 2015) and is involved in the SI response in *Olea europaea* (Solfanelli et al., 2006), *Petunia hybrida* (Kovaleva and Zakharova, 2003), and *Theobroma cacao* (Hasenstein and Zavada, 2001); ethylene/abscisic acid were also found to play a vital role in the pollen-pistil interaction in the SI of *Petunia* (Kovaleva and Zakharova, 2003). In addition, we found that the “ATP-binding cassette (ABC)” transporter B and G family protein participated in the HetSI of *P. auriculata.* ABCG and ABCB transporters were previously demonstrated to implicate in auxin transport (Balzan et al., 2014; Chang et al., 2016; Liu et al., 2020) for the regulation of male gametophyte development and self-incompatibility, particularly by facilitating pollen tube growth (Gao et al., 2019; Fu et al., 2023). From all above, we can infer that plant hormone plays a pivotal role in HetSI.

### 4.5 Lower energy consumption for SI pollination in HetSI

Pentose and glucuronate interconversion pathways and the citric acid cycle were energy sources for pollen tube growth. DEGs involved in the interconversions of pentose and glucuronate pathway was upregulated in SC (Table 4). In the TCA cycle (citrate cycle) pathways-related *pdhC* (pyruvate dehydrogenase E2 component), *MDH1* (Malate dehydrogenase), *pdhB* (pyruvate dehydrogenase E1 component subunit beta), and *ACLY* (ATP citrate (pro-S)-lyase) were dramatically downregulated in “T × P” vs. “T × T”; *MDH1*, *pdhC*, and *ACLY* exhibited consistent trends in the cross-comparison “P × T” vs. “P × P”. The aforementioned nutrients linked to energy need to be metabolized to create acetyl-CoA, which must brought into the cycle of TCA to enhance ATP generation to facilitate the growth of the pollen tube (Obermeyer et al., 2013). Moreover, pollen germination was deferred in comparison to “T × P” pollinations. The level of energy-related genes was more pronounced in “T × P” vs. “T × T”, and growing pollen tubes could also consume more supplements in “T × P” for faster pollen tube growth.

Fatty acids might play the same role in HetSI; they can provide a nutrient-rich environment for pollen hydration and germination, and the accumulation of fatty acids could abrogate the SI response (Qin et al., 2022). The degradation of fatty acids is an oxidative process channeled into gluconeogenesis via the glyoxylate cycle with concomitant energy release (Hernández et al., 2020). In the “T × P” vs. “T × T” comparison, *ACOX1* (acyl-CoA oxidase), *HADH* (3-hydroxyacyl-CoA dehydrogenase), *ECHA* (enoyl-CoA hydratase), and *HADHA* (enoyl-CoA hydratase) in the fatty acid degradation pathway were sharply downregulated. Additionally, *ACSL* (long-chain acyl-CoA synthetase) showed the same trend in the “P × T” vs. “P × P” comparison, which indicated lower energy needs in SI pollination (Supplementary Figure S4). Moreover, fatty acid degradation might also involve the constituents of surface lipid polymers (cutin and suberine) on the stigma (Heizmann et al., 2000). To facilitate the growth of the pollen tube, lipids assumed at high levels in SC pollinations in our metabolomic study (Hu et al., 2019). In the process of this study, lipid transport and metabolism were also enriched. Lipids play a crucial part in the pollen-stigma capture’s hydrophobic nature (Heizmann et al., 2000) and membrane signalling during the growth of the pollen tube (Potocký et al., 2003), which have a great influence on the pollen-pistil interaction of HetSI.

Flavonoids also serve as energy-related nutrients for pollen tube growth and impact sexual reproduction. According to Lan et al.’s (2017) research, flavonoid accumulation in stigmas could break the SI in *Brassica*. Flavonoids may also be involved in cell wall loosening on stigmas, which can facilitate SC pollen tube penetration (Wu et al., 2023). In our preliminary metabolic study, the overall content of flavonoids in SC significantly increased (Hu et al., 2019). In this study, we found a dramatic decline in *CHS* (Chalcone synthase), *CHI* (Chalcone isomerase), and *DFR* (Dihydroflavonol 4-reductase) in “T × P”vs. “T × T”, *FLS* (Flavanol synthase), *CHS* and *DFR* in “P × T” vs. “P × P”. These genes all play a significant regulatory role in the accumulation of flavonoids (Nakatsuka et al., 2005) and displayed a similar trend with metabolomic results.

## 5 Conclusion

Using high-throughput RNA-seq, we carried out a transcriptomic comparative investigation of SC and SI pollination in *Plumbago auriculata* in this research. The S-locus in *P. auriculata* HetSI was further identified. The SSI-related candidates *SRK*, *SRK6*, *PSEUDOSRKA*, *ARC1*, *MLPK*, *THL1/2* and *Exo70A1* and *MOD* were found involved in HetSI. In addition, *SI S1* was one of the key factors in GSI, however, it appeared with an opposite manner in HetSI. Comparing SC with SI, many notable genes potentially involved in HetSI were abundant in “MAPK (plant) signaling pathway”, “plant hormone signal transduction”, “plant-pathogen interaction”, and “pentose and glucuronate interconversions” KEGG pathways. Furthermore, we found that the gene expression of HetSI in different styles was distinct, and the HetSI mechanism seems more complicated in thrum morphotypes. These results offer complementary data for investigating the specific mechanism of HetSI. However, high-throughput techniques can only screen candidates involved in HetSI, molecular interactions in HetSI is still uncertain. Therefore, this research lays a basis for further exploration of molecular interactions in HetSI.

## Data Availability

The datasets presented in this study can be found in the Sequence Read Archive (SRA) Repository (https://www.ncbi.nlm.nih.gov/sra). Accession number PRJNA1074424.

## References

[B1] Abhinandan K. Hickerson N. M. Lan X. Samuel M. A. (2023). Disabling of ARC1 through CRISPR–Cas9 leads to a complete breakdown of self-incompatibility responses in *Brassica napus* . Plant Commun. 13 (2), 100504. 10.1016/j.xplc.2022.100504 PMC10030360 36518081

[B2] Abhinandan K. Sankaranarayanan S. Macgregor S. Goring D. R. Samue M. A. (2022). Cell–cell signaling during the Brassicaceae self-incompatibility response. Trends Plant Sci. 27, 472–487. 10.1016/j.tplants.2021.10.011 34848142

[B3] Balzan S. Johal G. S. Carraro N. (2014). The role of auxin transporters in monocots development. Front. Plant Sci. 5, 393. 10.3389/fpls.2014.00393 25177324 PMC4133927

[B4] Banović B. Miljuš-Đukić J. Konstantinović M. Maksimović V. (2010). A search of *Brassica* SI-involved orthologs in buckwheat leads to novel buckwheat sequence identification: MLPK possibly involved in SI response. Arch. Biol. Sci. 62, 315–321. 10.2298/ABS1002315B

[B5] Baumberger N. Doesseger B. Guyot R. Diet A. Parsons R. L. Clark M. A. (2003). Whole-genome comparison of leucine-rich repeat extensins in *Arabidopsis* and rice. A conserved family of cell wall proteins form a vegetative and a reproductive clade. Plant Phys. 131, 1313–1326. 10.1104/pp.102.014928 PMC166891 12644681

[B6] Benjamini Y. Hochberg Y. (1995). Controlling the false discovery rate: a practical and powerful approach to multiple testing. J. R. Stat. Soc. B (Methodol.) 57, 289–300. 10.1111/j.2517-6161.1995.tb02031.x

[B7] Cabrillac D. Cock J. M. Dumas C. Gaude T. (2001). The S-locus receptor kinase is inhibited by thioredoxins and activated by pollen coat proteins. Nature 410, 220–223. 10.1038/35065626 11242083

[B8] Catanzariti A. M. Lim G. T. T. Jones D. A. (2015). The tomato *I-3* gene: a novel gene for resistance to *Fusarium* wilt disease. New Phytol. 207, 106–118. 10.1111/nph.13348 25740416

[B9] Chang Z. Chen Z. Yan W. Xie G. Lu J. Wang N. (2016). An ABC transporter, OsABCG26, is required for anther cuticle and pollen exine formation and pollen-pistil interactions in rice. Plant Sci. 253, 21–30. 10.1016/j.plantsci.2016.09.006 27968990

[B11] Doucet J. Lee H. K. Goring D. R. (2016). Pollen acceptance or rejection: a tale of two pathways. Trends Plant Sci. 21, 1058–1067. 10.1016/j.tplants.2016.09.004 27773670

[B12] Finnegan E. J. Liang D. Wang M. B. (2011). Self-incompatibility: *Smi* silences through a novel sRNA pathway. Trends Plant Sci. 16 (5), 238–241. 10.1016/j.tplants.2011.01.002 21306936

[B13] Fu Y. Zhang H. Ma Y. Li C. Zhang K. Liu X. (2023). A model worker: multifaceted modulation of AUXIN RESPONSE FACTOR3 orchestrates plant reproductive phases. Front. Plant Sci. 14, 1123059. 10.3389/fpls.2023.1123059 36923132 PMC10009171

[B14] Fujii S. Shimosato-Asano H. Kakita M. Kitanishi T. Iwano M. Takayama S. (2020). Parallel evolution of dominant pistil-side self-incompatibility suppressors in *Arabidopsis* . Nat. Commun. 11, 1404. 10.1038/s41467-020-15212-0 32179752 PMC7075917

[B15] Gao C. Wang Y. Qu H. (2019). Study of auxin regulation of pollen tube growth through calcium channels in *Pyrus pyrifolia* . Plant Growth Regul. 89, 99–108. 10.1007/s10725-019-00522-1

[B16] Goring D. R. Bosch M. Franklin-Tong V. E. (2023). Contrasting self-recognition rejection systems for self-incompatibility in *Brassica* and *Papaver* . Curr. Biol. 33, R530–R542. 10.1016/j.cub.2023.03.037 37279687

[B17] Goring D. R. Indriolo E. Samuel M. A. (2014). The ARC1 E3 ligase promotes a strong and stable self-incompatibility response in *Arabidopsis* species: response to the *Nasrallah* and *Nasrallah* commentary. Plant Cell 26, 3842–3846. 10.1105/tpc.114.131243 25336510 PMC4247588

[B18] Gu T. Mazzurco M. Sulaman W. Matias D. D. Goring D. R. (1998). Binding of an arm repeat protein to the kinase domain of the S-locus receptor kinase. Proc. Natl. Acad. Sci. U. S. A. 95, 382–387. 10.1073/pnas.95.1.382 9419384 PMC18231

[B19] Hasenstein K. H. Zavada M. S. (2001). Auxin modification of the incompatibility response in *Theobroma cacao* . Physiol. Plant. 112, 113–118. 10.1034/j.1399-3054.2001.1120115.x 11319022

[B20] Heizmann P. Luu D. T. Dumas C. (2000). Pollen-stigma adhesion in the *Brassicaceae* . Ann. Bot. 85, 23–27. 10.1006/anbo.1999.1057

[B21] Hernández M. L. Lima-Cabello E. Alché J. D. D. Martínez-Rivas J. M. Castro A. J. (2020). Lipid composition and associated gene expression patterns during pollen germination and pollen tube growth in olive (*Olea europaea* L.). Plant Cell Physiol. 61, 1348–1364. 10.1093/pcp/pcaa063 32384163 PMC7377348

[B22] Hou S. Zhao T. Yang Z. Yang D. Li Q. Liang L. (2022). Molecular cloning and yeast two-hybrid provide new evidence for unique sporophytic self-incompatibility system of *Corylus* . Plant Biol. J. 24, 104–116. 10.1111/plb.13347 34724309

[B23] Hu D. Gao S. Li W. Lei T. Liu Y. Li Q. (2020). Dissecting the distally response to pollination using metabolite profiling in heteromorphic incompatibility system interactions of *Plumbago auriculata* Lam. Acta Physiol. Plant. 42, 122. 10.1007/s11738-020-03111-2

[B24] Hu D. Li W. Gao S. Lei T. Hu J. Shen P. (2019). Untargeted metabolomic profiling reveals that different responses to self and cross pollination in each flower morph of the heteromorphic plant *Plumbago auriculata* . Plant Physiol. biochem. 144, 413–426. 10.1016/j.plaphy.2019.10.010 31634809

[B25] Huang J. Yang L. Yang L. Wu X. Cui X. Zhang L. (2023). Stigma receptors control intraspecies and interspecies barriers in Brassicaceae. Nature 614, 303–308. 10.1038/s41586-022-05640-x 36697825 PMC9908550

[B26] Huu C. N. Plaschil S. Himmelbach A. Kappel C. Lenhard M. (2022). Female self-incompatibility type in heterostylous *Primula* is determined by the brassinosteroid-inactivating cytochrome P450 CYP734A50. Curr. Biol. 32, 671–676.e5. 10.1016/j.cub.2021.11.046 34906354

[B27] Jain S. Maurya P. Jain S. Kumar V. Amulya S. Kiran B. (2023). Incompatibility systems in fruit crops: applications and achievements. Int. J. Environ. Clim. Chang. 13, 2653–2663. 10.9734/IJECC/2023/v13i92496

[B28] Jamshed M. Sankaranarayanan S. Abhinandan K. Samuel A. M. (2020). Stigma receptivity is controlled by functionally redundant MAPK pathway components in *Arabidopsis* . Molec. Plant 13, 1582–1593. 10.1016/j.molp.2020.08.015 32890733

[B29] Kakita M. Murase K. Iwano M. Matsumoto T. Watanabe M. Shiba H. (2007b). Two distinct forms of M-locus protein kinase localize to the plasma membrane and interact directly with S-locus receptor kinase to transduce self-incompatibility signaling in *Brassica rapa* . Plant Cell 19, 3961–3973. 10.1105/tpc.106.049999 18065692 PMC2217645

[B30] Kakita M. Shimosato H. Murase K. Isogai A. Takayama S. (2007a). Direct interaction between S-locus receptor kinase and M-locus protein kinase involved in *Brassica* self-incompatibility signaling. Plant Biotechnol. 24, 185–190. 10.5511/plantbiotechnology.24.185

[B31] Kitashiba H. Nasrallah J. B. (2014). Self-incompatibility in Brassicaceae crops: lessons for interspecific incompatibility. Breed. Sci. 64, 23–37. 10.1270/jsbbs.64.23 24987288 PMC4031107

[B32] Kovaleva L. Zakharova E. (2003). Hormonal status of the pollen-pistil system at the progamic phase of fertilization after compatible and incompatible pollination in *Petunia hybrida* L. Sex. Plant Reprod. 16, 191–196. 10.1007/s00497-003-0189-1

[B33] Lan X. Yang J. Kumar A. Nie Y. Li X. Li Y. (2017). Flavonoids and ROS play opposing roles in mediating pollination in ornamental kale (*Brassica oleracea* var. acephala). Mol. Plant. 10, 1361–1364. 10.1016/j.molp.2017.08.002 28827168

[B34] Li J. Li P. Li J. Zhang L. Zhang X. (2023). Morphological characteristics and molecular markers of distally in *Primula obconica* . Euphytica 219, 72. 10.1007/s10681-023-03198-x

[B35] Li M. Xu W. Yang W. Kong Z. Xue Y. (2007). Genome-wide gene expression profiling reveals conserved and novel molecular functions of the stigma in rice. Plant Physiol. 144, 1797–1812. 10.1104/pp.107.101600 17556504 PMC1949881

[B36] Li Z. He Y. (2020). Roles of brassinosteroids in plant reproduction. Int. J. Mol. Sci. 21, 872. 10.3390/ijms21030872 32013254 PMC7037687

[B37] Lian X. Zhang S. Huang G. Huang L. Zhang J. Hu F. (2021). Confirmation of a gametophytic self-incompatibility in *Oryza longistaminata* . Oryza Longistaminata. Front. Plant. Sci. 12, 576340. 10.3389/fpls.2021.576340 33868321 PMC8044821

[B38] Liu L. Zhao L. Chen P. Cai H. Hou Z. Jin X. (2020). ATP binding cassette transporters ABCG1 and ABCG16 affect reproductive development via auxin signalling in *Arabidopsis* . Plant J. 102 (6), 1172–1186. 10.1111/tpj.14690 31944421

[B39] Livak K. J. Schmittgen T. D. (2001). Analysis of relative gene expression data using real-time quantitative PCR and the 2^−ΔΔCT^ method. Methods 25, 402–408. 10.1006/meth.2001.1262 11846609

[B40] Lu W. Bian X. Yang W. Cheng T. Wang J. Zhang Q. (2018). Transcriptomics investigation into the mechanisms of self-incompatibility between pin and thrum morphs of *Primula maximowiczii* . Int. J. Mol. Sci. 19, 1840. 10.3390/ijms19071840 29932122 PMC6073747

[B41] Lv S. Qiao X. Zhang W. Li Q. Wang P. Zhang S. (2022). The origin and evolution of RNase T2 family and gametophytic self-incompatibility system in plants. Genome Biol. Evol. 14, evac093. 10.1093/gbe/evac093 35714207 PMC9250077

[B42] Matsuda T. Matsushima M. Nabemoto M. Osaka M. Sakazono S. Masuko-Suzuki H. (2015). Transcriptional characteristics and differences in *Arabidopsis* stigmatic papilla cells pre- and post-pollination. Plant Cell Physiol. 56, 663–673. 10.1093/pcp/pcu209 25527828

[B43] Matsui K. Yasui Y. (2020). Buckwheat heteromorphic self-incompatibility: Genetics, genomics and application to breeding. Breed. Sci. 70, 32–38. 10.1270/jsbbs.19083 32351302 PMC7180150

[B44] Maurel C. Verdoucq L. Luu D. T. Santoni V. (2008). Plant aquaporins: membrane channels with multiple integrated functions. Annu. Rev. Plant Biol. 59, 595–624. 10.1146/annurev.arplant.59.032607.092734 18444909

[B45] McCubbin A. (2008). “Heteromorphic self-incompatibility in primula: twenty-first century tools promise to unravel a classic nineteenth century model system,” in Self-incompatibility in flowering plants: evolution, diversity, and mechanisms. Editor Franklin-Tong V. E. (Berlin, Heidelberg: Springer), 289–308.

[B46] Miao H. Ye Z. Hu G. Qin Y. (2015). Comparative transcript profiling of gene expression between self-incompatible and self-compatible mandarins by suppression subtractive hybridization and cDNA microarray. Mol. Breed. 35, 47. 10.1007/s11032-015-0204-x

[B47] Mucina L. Hammer T. (2019). *Limonium dagmarae* (*Plumbaginaceae*), a new species from Namaqualand coast, South Africa. Phytotaxa 403, 71–85. 10.11646/phytotaxa.403.2.1

[B48] Muñoz-Sanz J. V. Zuriaga E. Cruz-García F. McClure B. Romero C. (2020). Self-(in)compatibility systems: target traits for crop-production, plant breeding, and biotechnology. Front. Plant Sci. 11, 195. 10.3389/fpls.2020.00195 32265945 PMC7098457

[B49] Murase K. Shiba H. Iwano M. Che F. S. Watanabe M. Isogai A. (2004). A membrane-anchored protein kinase involved in *Brassica* self-incompatibility signaling. Science 303, 1516–1519. 10.1126/science.1093586 15001779

[B50] Nakatsuka T. Nishihara M. Mishiba K. Yamamura S. (2005). Temporal expression of flavonoid biosynthesis-related genes regulates flower pigmentation in gentian plants. Plant Sci. 168, 1309–1318. 10.1016/j.plantsci.2005.01.009

[B51] Obermeyer G. Fragner L. Lang V. Weckwerth W. (2013). Dynamic adaption of metabolic pathways during germination and growth of lily pollen tubes after inhibition of the electron transport chain. Plant Physiol. 162, 1822–1833. 10.1104/pp.113.219857 23660836 PMC3729764

[B52] Pauline M. Servane B. Corentin D. Francisco C. Juan-Pablo M. Stanley L. (2023). Intra- and inter-specific reproductive barriers in the tomato clade. Front. Plant. Sci. 14, 1326689. 10.3389/fpls.2023.1326689 38143584 PMC10739309

[B53] Potocký M. Eliás M. Profotová B. Novotná Z. Valentová O. Zárský V. (2003). Phosphatidic acid produced by phospholipase D is required for tobacco pollen tube growth. Planta 217, 122–130. 10.1007/s00425-002-0965-4 12721856

[B54] Qin H. Li H. Abhinandan K. Xun B. Yao K. Shi J. (2022). Fatty acid biosynthesis pathways are downregulated during stigma development and are critical during self-incompatible responses in *ornamental kale* . Int. J. Mol. Sci. 23, 13102. 10.3390/ijms232113102 36361887 PMC9656282

[B55] Sanabria N. Goring D. Nürnberger T. Dubery I. (2008). Self/nonself perception and recognition mechanisms in plants: a comparison of self-incompatibility and innate immunity. New Phytol. 178, 503–514. 10.1111/j.1469-8137.2008.02403.x 18346103

[B56] Sassa H. (2016). Molecular mechanism of the S-RNase-based gametophytic self-incompatibility in fruit trees of *Rosaceae* . Breed. Sci. 66, 116–121. 10.1270/jsbbs.66.116 27069396 PMC4780795

[B57] Shi D. Huang H. Zhang Y. Qian Z. Du J. Huang L. (2024). The roles of non-coding RNAs in male reproductive development and abiotic stress responses during this unique process in flowering plants. Plant Sci. 341, 111995. 10.1016/j.plantsci.2024.111995 38266717

[B58] Smith D. K. Jones D. M. Lau J. B. R. Cruz E. R. Brown E. Harper J. F. (2018). A putative protein o-Fucosyltransferase facilitates pollen tube penetration through the stigma-style interface. Plant Phys. 176, 2804–2818. 10.1104/pp.17.01577 PMC5884604 29467178

[B59] Solfanelli C. Bartolini S. Vitagliano C. Lorenzi R. (2006). Immunolocalization and quantification of IAA after self- and free-pollination in *Olea europaea* L. Sci. Hortic. 110, 345–351. 10.1016/j.scienta.2006.06.026

[B60] Takasaki T. Hatakeyama K. Suzuki G. Watanabem M. Isogai A. Hinata K. (2000). The S receptor kinase determines self-incompatibility in *Brassica* stigma. Nature 403, 913–916. 10.1038/35002628 10706292

[B61] Takayama S. Shiba H. Iwano M. Shimosato H. Che F. S. Kai N. (2000). The pollen determinant of self-incompatibility in *Brassica campestris* . Proc. Natl. Acad. Sci. U. S. A. 97, 1920–1925. 10.1073/pnas.040556397 10677556 PMC26537

[B62] Tang C. Zhu X. Qiao X. Gao H. Li Q. Wang P. (2020). Characterization of the pectin methyl-esterase gene family and its function in controlling pollen tube growth in pear (*Pyrus bretschneideri*). Genomics 112, 2467–2477. 10.1016/j.ygeno.2020.01.021 32014523

[B63] Wang F. Li Y. Li G. Cheng S. (2023). Genetic components of self-incompatibility in brassica vegetables. Horticulturae 9, 265. 10.3390/horticulturae9020265

[B64] Wu H. M. Xie D. J. Jia P. F. Tang Z. S. Shi D. Q. Shui G. H. (2023). Homeostasis of flavonoids and triterpenoids most likely modulates starch metabolism for pollen tube penetration in rice. Plant Biotechnol. J. 21, 1757–1772. 10.1111/pbi.14073 37221659 PMC10440988

[B65] Wu Y. Yan J. Zhang R. Qu X. Huang S. Chen N. (2010). *Arabidopsis* FIMBRIN5, an actin bundling factor, is required for pollen germination and pollen tube growth. Plant Cell 22, 3745–3763. 10.1105/tpc.110.080283 21098731 PMC3015131

[B66] Yang Q. Fu Y. Zhang T. Peng S. Deng J. (2022). Identification of microRNAs related to phytohormone signal transduction and self-incompatibility of rabbiteye blueberry pollen. J. Am. Soc. Hortic. 147 (6), 300–311. 10.21273/JASHS05143-21

[B67] Zhang J. Yue L. Wu X. Liu H. Wang W. (2021a). Function of small peptides during male-female crosstalk in plants. Front. Plant Sci. 12, 671196. 10.3389/fpls.2021.671196 33968121 PMC8102694

[B68] Zhang Z. Zhan H. Lu J. Xiong S. Yang N. Yuan H. (2021b). Tapetal 3-Ketoacyl-aoenzyme a synthases are involved in pollen coat lipid accumulation for pollen-stigma interaction in *Arabidopsis* . Front. Plant Sci. 12, 770311. 10.3389/fpls.2021.770311 34887893 PMC8650583

[B69] Zhao P. Zhang L. Zhao L. (2015). Dissection of the style's response to pollination using transcriptome profiling in self-compatible (*Solanum pimpinellifolium*) and self-incompatible (*Solanum chilense*) tomato species. BMC Plant Biol. 15, 119. 10.1186/s12870-015-0492-7 25976872 PMC4431037

[B70] Zhou J. Liang Y. Niu Q. Chen L. Zhang X. Ye D. (2013). The *Arabidopsis* general transcription factor TFIIB1 (AtTFIIB1) is required for pollen tube growth and endosperm development. J. Exp. Bot. 8, 2205–2218. 10.1093/jxb/ert078 PMC3654413 23547107

[B71] Zhou J. Lu M. Yu S. Liu Y. Yang J. Tan X. (2020). In-depth understanding of *Camellia oleifera* self-incompatibility by comparative transcriptome, proteome and metabolome. Int. J. Mol. Sci. 21, 1600. 10.3390/ijms21051600 32111089 PMC7084461

