# Supplementary figures and images for: Comparative stigmatic transcriptomics reveals self and cross pollination responses to heteromorphic incompatibility in Plumbago auriculata Lam
Source: Front Genet. 2024 Mar 6;15:1372644. doi: 10.3389/fgene.2024.1372644 (PMC10953596; doi:10.3389/fgene.2024.1372644)

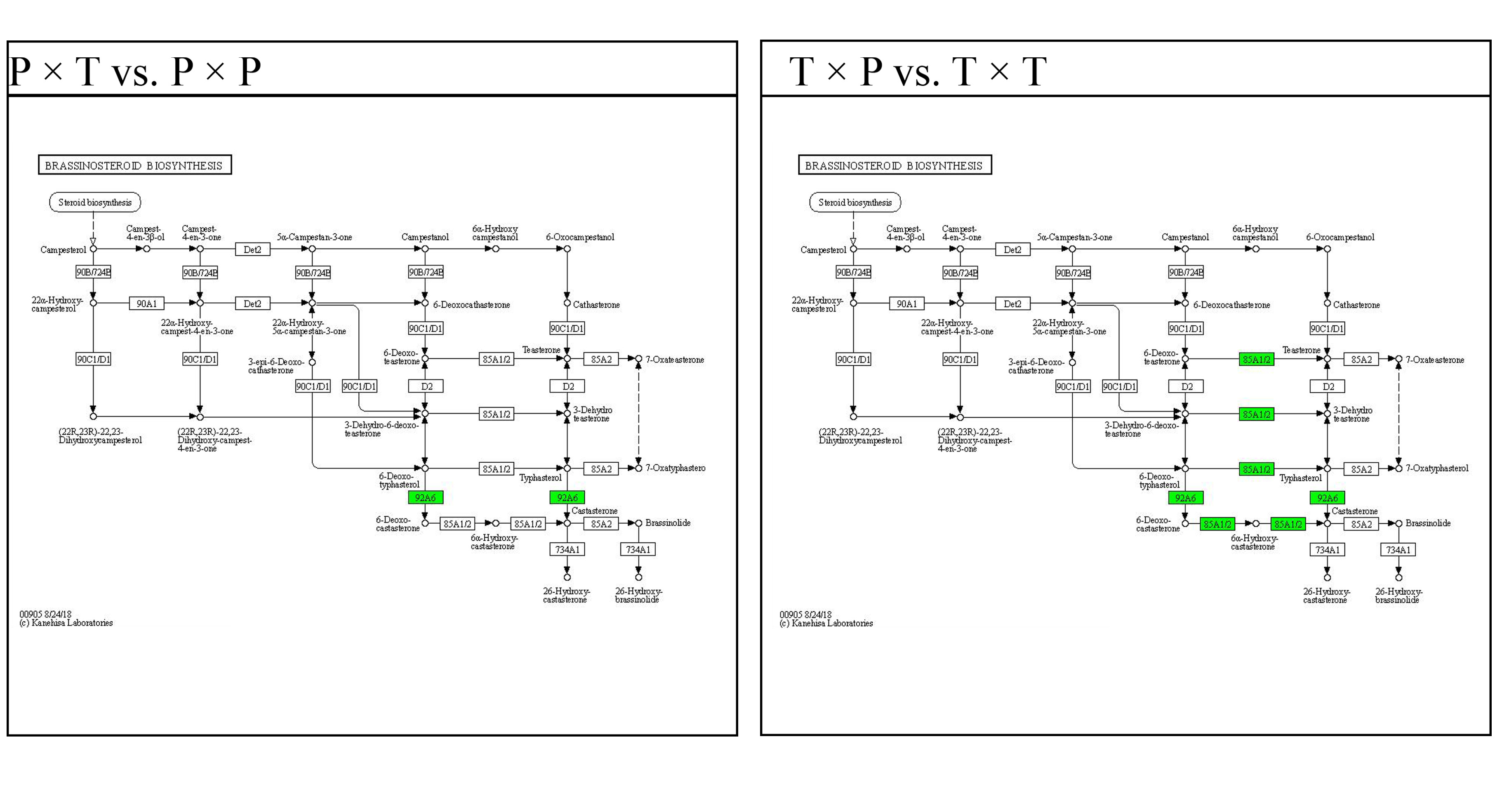

Supplement: Supplementary file 2 [file Image3.JPEG]

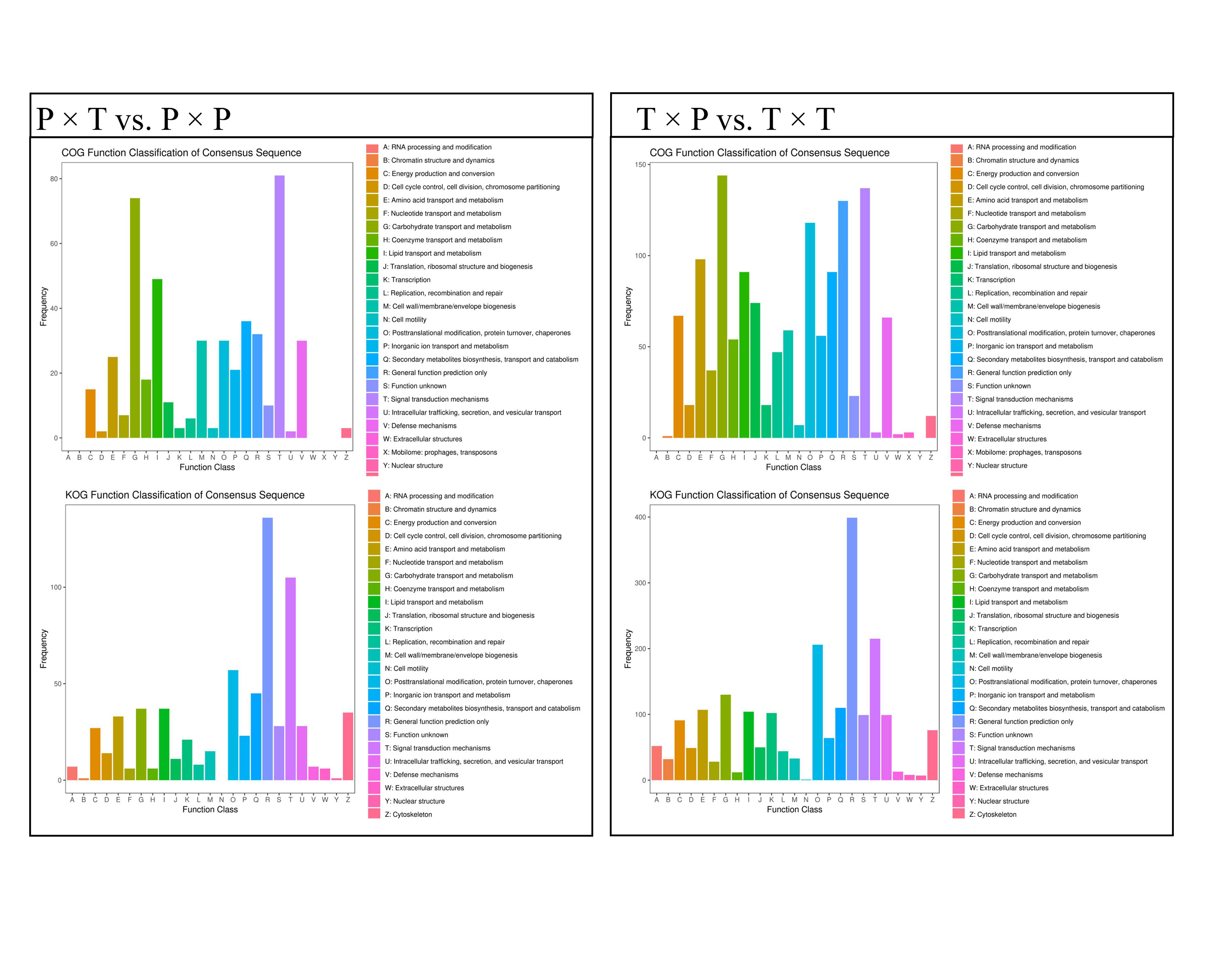

Supplement: Supplementary file 4 [file Image1.JPEG]

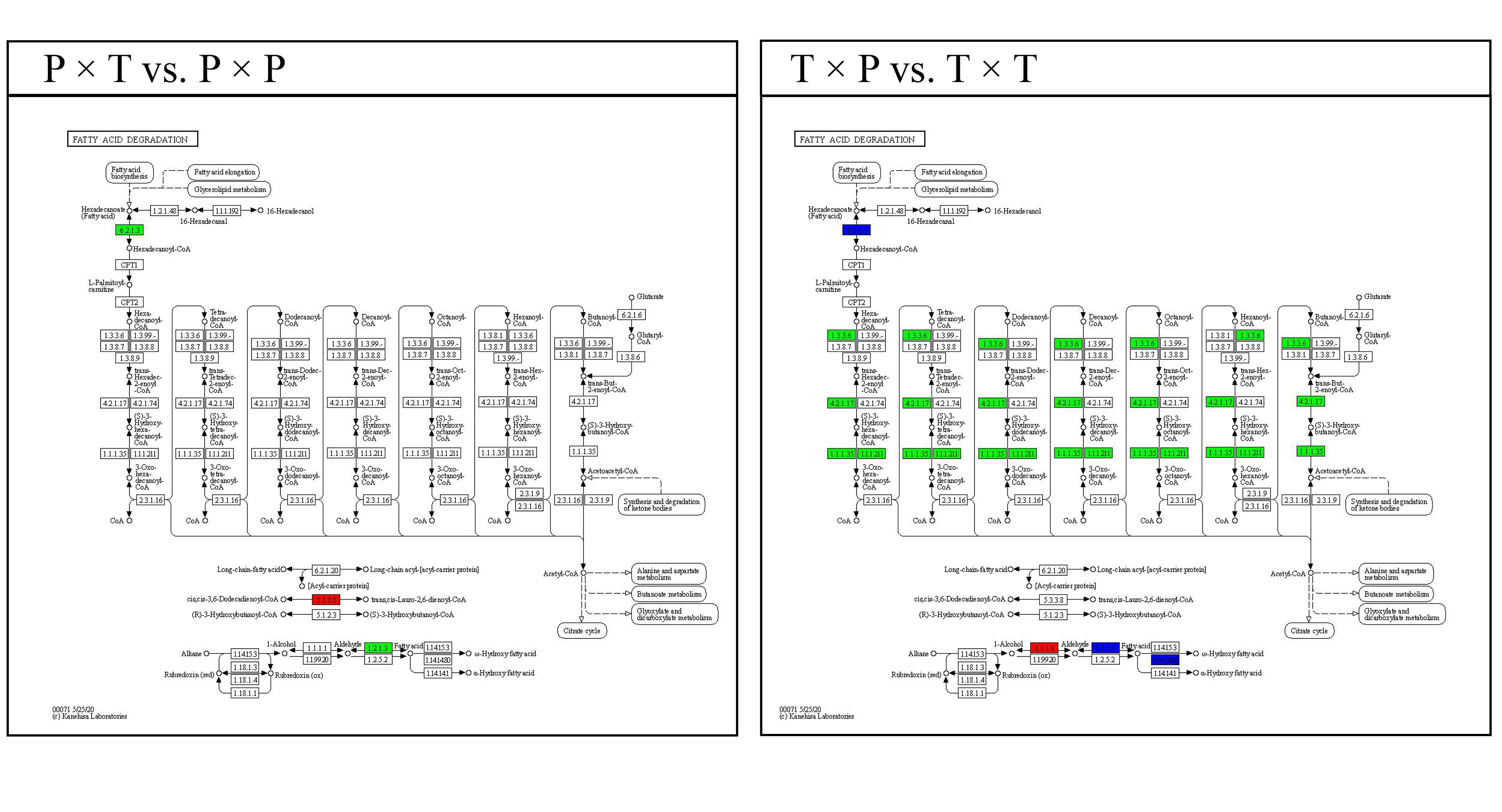

Supplement: Supplementary file 5 [file Image4.JPEG]

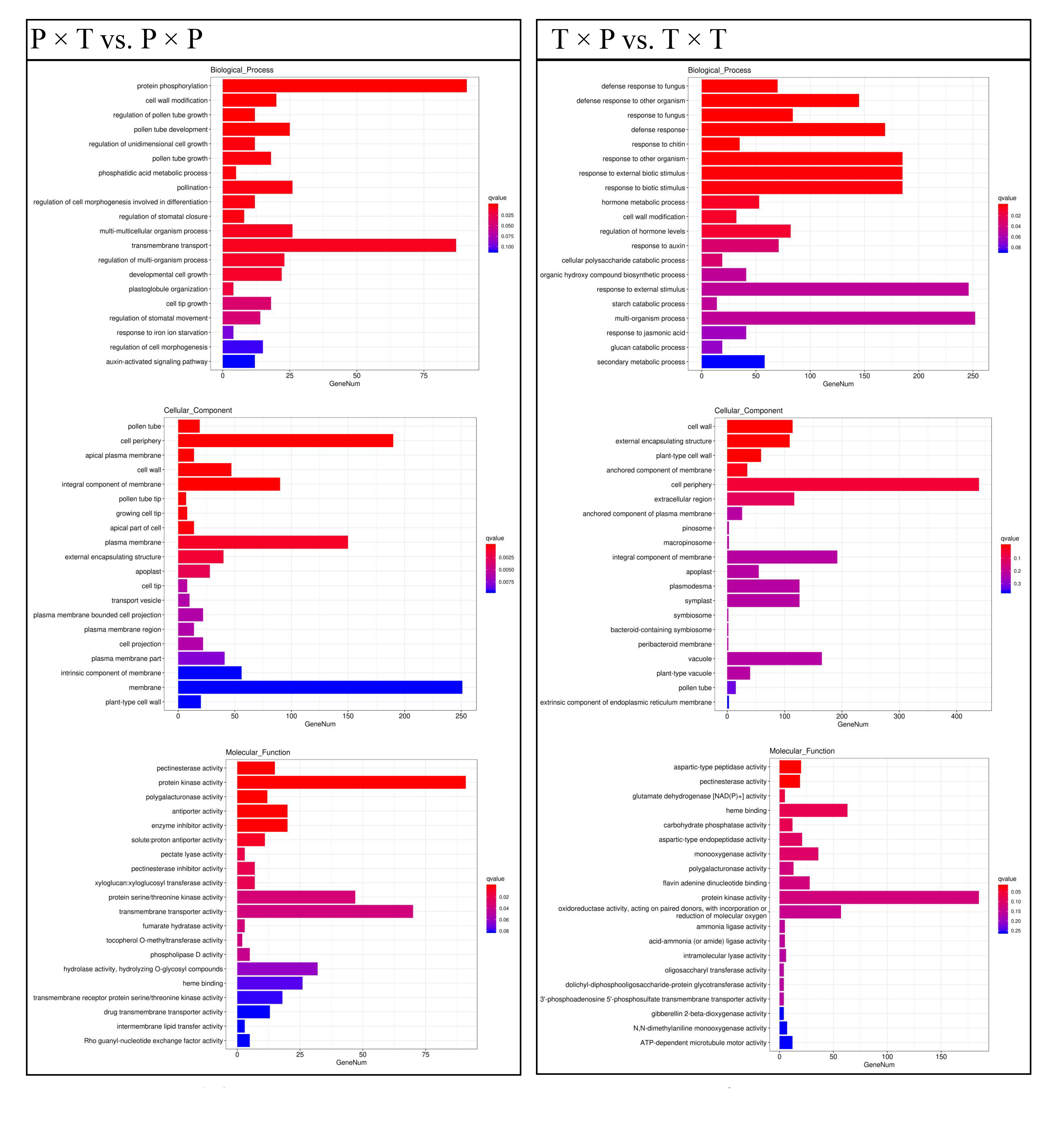

Supplement: Supplementary file 6 [file Image2.JPEG]
